# Does prenatal micronutrient supplementation improve children's mental development? A systematic review

**DOI:** 10.1186/1471-2393-11-12

**Published:** 2011-02-03

**Authors:** Brenda MY Leung, Kristin P Wiens, Bonnie J Kaplan

**Affiliations:** 1 Dept. of Community Health Sciences, Faculty of Medicine, University of Calgary, 3330 Hospital Drive NW, Calgary, AB, T2N 4N1, Canada; 2 Dept. of Pediatrics, University of Calgary, Behaviour Research Unit, Alberta Children's Hospital, 2888 Shaganappi Trail NW, Calgary, AB, T3B 6A8, Canada

## Abstract

**Background:**

Although maternal nutrient status influences all aspects of fetal development including the brain, the impact of micronutrient supplementation on the baby's mental function is a topic of debate. This systematic review assesses the effect of single and multiple micronutrient supplementation during pregnancy on offspring mental development.

**Methods:**

Eleven electronic literature databases were searched using key terms of various combinations and filter string terms. Reference lists of articles selected for review were scanned for citations fitting the same inclusion criteria. Each stage of the literature retrieval and review process was conducted independently by two reviewers. The CONSORT checklist was used to assess study quality.

**Results:**

A total of 1316 articles were retrieved from the electronic database search, of which 18 met the inclusion criteria and were evaluated. The selected studies were randomized controlled trials published from 1983 to 2010, with high variance in sample size, intervention type, and outcome measures. The median CONSORT score was 15 (range 12 - 19). Due to inconsistent interventions and outcome measures among the studies, no conclusive evidence was found that enhancing the intrauterine environment through micronutrient supplementation was associated with child mental development in a number of dimensions. There was some evidence to support n-3 fatty acids or multi-micronutrients having some positive effect on mental development, but the evidence for single nutrients was much weaker.

**Conclusions:**

The study of children's mental outcomes as a function of prenatal supplementation is still relatively new, but the results of this systematic review suggest that further work with multiple micronutrients and/or n-3 fatty acids should be conducted.

## Background

Almost universally, governments and health professionals suggest that pregnant women take prenatal formulas containing various micronutrients to ensure a healthy pregnancy and healthy baby. In addition to these general guidelines, the more specific use of B-vitamins (especially folic acid) is now commonly recommended, as they reduce the risk of neural tube defects if consumed during the periconceptional period. The impact of such perinatal supplementation is well described for variables involving physical health, including birth outcomes, fetal growth, and infant physical development [1,2]. For instance, a review by Shah et al. [3] concluded that pregnant women supplemented with multi-micronutrients had significantly lower risk for low birth weight (though not for prematurity) compared to women supplemented with only iron and folic acid.

Guidelines for prenatal supplementation are of considerable importance beyond the immediate obstetrical implications. The long-lasting health impact of the intrauterine environment on the developing foetus has become an area of investigation ever since Barker suggested the link for cardiovascular disease and type II diabetes [4]. One well-recognized component of the intrauterine environment that is likely responsible for many of those health effects is nutrition. It is now understood that maternal nutrient status can influence fetal development in all phases, including brain development, which would affect behaviour and cognitive function [5]. The support for an association between gestational nutrition and brain development has been particularly strong for iron, n-3 fatty acids, and folate [6-8].

In summary, there is no debate about the contribution of micronutrients to health, nor the importance of pregnant women eating well to maximize the outcomes for their babies. Even the concept of micronutrient supplementation for pregnant women appears to be a universally accepted practice, and is widely accepted as 'insurance' to prevent adverse perinatal outcomes, especially in those at risk for inadequate nutrient status due to other health factors [9]. However, the two topics that are still open to much investigation and debate are the optimal content of micronutrient supplementation, and whether there is a long-term impact on the baby's mental function.

The existing literature was systematically reviewed to assess the impact of prenatal supplementation on offspring mental development, including cognitive development, psychomotor abilities, intelligence, and behavior/temperament. This systematic review focused specifically on the effect of single and multiple micronutrient supplementation during pregnancy on offspring mental development.

## Methods

### Search Strategy

The search strategy selected randomized controlled trials (RCTs) and cohort studies in humans, with English-only text, with no limitations set for date of publication. BL consulted a Research Librarian at the Centre for Health and Policy Studies, Community Health Sciences at the University of Calgary to develop the search strategy, the inclusion/exclusion criteria, abstract screening tool, keyword list, validated search filters, and databases.

Eleven electronic literature databases were searched by BL between 22 - 30 Dec. 2009: Medline/PubMed (1950 to November Week 3 2009), HealthStar (1966 to Nov. 2009), EMBASE (1980 to 2009 Week 52), PsychInfo (1967 to Dec. week 4, 2009), CAB Nutrition Abstracts (1973 to 2009 week 51), Cochrane Library (1991 to Nov. 2009), AMED (1985 to December 2009), ERIC (1965 to Nov. 2009), CINAHL (to week of Dec. 22, 2009), Scopus and Web of Science (to week of Dec. 22, 2009).

The search syntax included four key parts: 1) terms defining the population of interest (pregnancy, pregnant women, prenatal, perinatal, antenatal); 2) terms for micronutrients (supplement, micronutrient, dietary supplement, vitamin, mineral, folic acid/folate, iron, iodine, B complex, B_12_, selenium, zinc, vitamin A, vitamin D); 3) terms for developmental outcomes (infant development, child development, mental development, brain development, neurodevelopment, cognitive development, psychomotor, IQ, behavior); and 4) terms for study design (RCT, pseudo-experimental, clinical trial, longitudinal cohort). Validated filters were used in the search strategy to ensure that all possible design terms for RCTs and cohort studies were used. The Cochrane search filters were used for RCTs [10] and the BMJ Knowledge filters were used for observational studies [11,12].

In addition, reference lists of articles selected for review were scanned for citations fitting the same inclusion criteria. This process enabled the identification of additional literature that may otherwise have been missed in the database search.

An updated search was conducted August 9, 2010 to look for articles published since the initial search. One study was found [13] and was included in the review.

### Inclusion Criteria

RCTs that investigated the effects of single or multi-micronutrient supplementation during pregnancy on child development (including mental, cognitive, psychomotor, intelligence and behavior) were included. Other study designs such as pseudo-experimental and cohort studies were excluded because only one of each of the aforementioned designs was found and the application of the CONSORT scoring (see section below on quality appraisal) was inappropriate for comparison to RCTs. Also, those reporting maternal nutrient intake or status (but not supplementation) and the effects on pregnancy or birth outcomes, and physical (but not mental) development or growth were excluded.

Identified citations were assessed against the inclusion criteria independently by two reviewers (BL and BK), first using the titles, then using the abstracts, and finally using the full text where there was disagreement. Seven articles were not in agreement between the two reviewers. Disagreement was resolved by discussion with re-examination of the document, and a consensus was reached. Following discussions, six of those seven were excluded. Articles on visual development were included after a review of the abstracts revealed that it was being used as a measure of neural development.

### Data Extraction and Quality Appraisal

Details of the studies were extracted by two reviewers (BL and KW) and summarized in tables. Key data elements extracted included subject and intervention characteristics, and outcomes of interest. Data from each accepted study were reviewed and extracted independently by BL and KW; quality assessment was determined by using the revised CONSORT 25-item checklist [14]. Consensus score was used for all analyses. The intraclass correlation coefficient for inter-observer agreement was calculated. Four CONSORT items were excluded because they were not applicable (19 (Harms), and 23 - 25 (Other information)), leaving a maximum score of 21 for each study (one point for each CONSORT item satisfied). The items were not weighted because the CONSORT statement is not a validated instrument. The articles were rated "good" quality if the score was ≥ 17 (meeting >80% of the checklist items), "average" if the score was between 13 - 16 (meeting 60-79% of the checklist items) and "poor" if ≤ 12 (meeting <59% of the checklist items).

## Results

**Figure 1:**
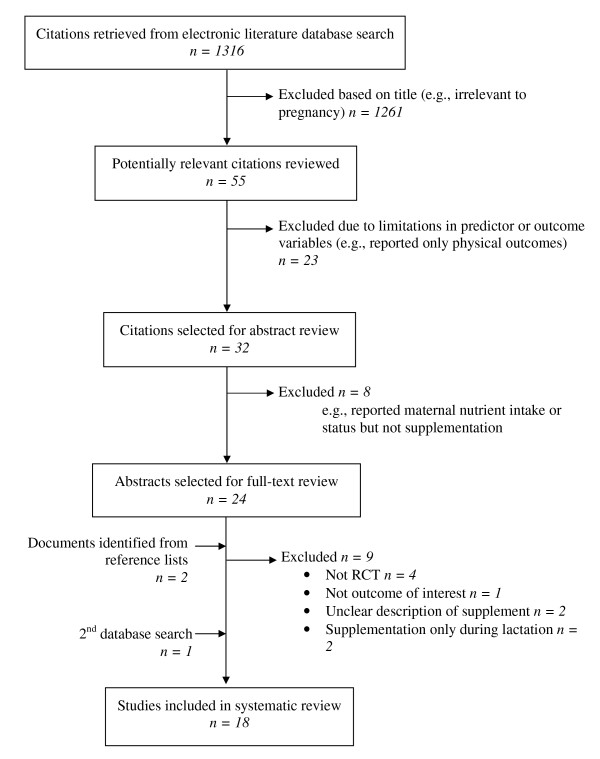
**Selection of studies for systematic review of the effect of prenatal supplementation with micronutrients on infant/child development**.

A total of 1316 articles were retrieved from the electronic database search, of which 18 met the inclusion criteria and were included in the final review (Figure 1). Three trials reported only on cognitive development [15-17], three reported visual development outcomes [18-20], one reported behavior and temperament alone [21], and the remaining studies examined outcomes for cognitive, psychomotor, behavior, visual and/or auditory development (Table 1) in various combinations. The outcome measure most widely used was the Bayley Scales of Infant Development (seven studies) [22-28].

**Table 1:** Summary of measures, results, and quality scores of articles included in systematic review

Reference	Sample	Outcomes	Measures	Intervention/Duration	Results	Quality Score
Caulfield et al. [13]	184 children (Peru)	1,3 (At 4-5 yrs)	Wechsler Preschool & Primary Scale of Intelligence	Zinc + folic acid + iron vs. folic acid + iron only 10-14 weeks gestation to birth	∅ 1,3	15/21
						
			Goodenough & Harris Draw-a-Person Test			
						
			Vineland Adaptive Behaviour Scales			
						
			Preschool Behaviour Questionnaire			

Dunstan et al. [31]	72 children (Australia)	1,2,3 (At 34 mo)	Griffiths Mental Development Scale	Fish oil vs. olive oil 20 weeks gestation to birth	∅ 1,3 + 2	16/21
						
			Peabody Picture Vocabulary Test			
						
			Child Behavior Checklist			

Hamadani et al. [22]	168 children (Bangladesh)	1,2,3 (At 39 mo)	Bayley Scales of Infant Development	Zinc vs. placebo 16 weeks gestation to birth	- 1,2 ∅ 3	14/21
						
			Wolke's Behaviour Rating Scale (modified)			

Helland et al. [16]	84 children (Norway)	1 (At 4 yrs)	Kaufman Assessment Battery for Children	Cod liver oil vs. corn oil 18 weeks gestation to three months post-birth	+ 1	12/21

Helland et al. [15]	143 children (Norway)	1 (At 7 yrs)	Kaufman Assessment Battery for Children	Cod liver oil vs. corn oil 18 weeks gestation to three months post-birth	∅ 1	15/21
						
Innis & Friesen [19]	135 infants (British Columbia)	4 (At 60 d)	Teller Acuity Card Procedure	DHA vs. corn/soybean oil 16 weeks gestation to birth	∅ 4	16/21

Joos et al. [25]	99 infants (rural Taiwan)	1,2 (At 250 d)	Bayley Scales of Infant Development	HCHP drink + MVM vs. LCLP drink + MVM Preconception to lactation	∅ 1 + 2	15/21

Judge et al. [18]	30 infants (Connecticut)	4 (At 4 and 6 mo)	Teller Acuity Card Procedure	DHA vs. corn oil cereal bar 24 weeks gestation to birth	+ 4 at 4 months ∅ 4 at 6 months	14/21

Judge et al. [17]	29 infants (Connecticut)	1 (At 9 mo)	Willatts' Infant Planning Test**^a^**	DHA vs. corn oil cereal bar 24 weeks gestation to birth	+ 1**^a^** ∅ 1**^b^**	12/21
						
			Fagan Test of Intelligence**^b^**			

Li et al. [26]	1305 infants (rural China)	1,2 (At 3,6,12 mo)	Bayley Scales of Infant Development	MVM vs. folic acid + iron vs. folic acid alone 14 weeks gestation to birth	+ 1 at 12 months ∅ 1 at 3 and 6 months ∅ 2	19/21

Malcolm et al. [20]	55 infants (Scotland)	4 (At 50 and 66 wk)	Visual evoked potential	Fish oil vs. sunflower oil 15 weeks gestation to birth	∅ 4	15/21

McGrath et al. [27]	327 children born to HIV-infected mothers (Tanzania)	1,2 (At 6, 12 and 18 mo)	Bayley Scales of Infant Development	Vitamin A vs. MVM-A vs. MVM+A vs. placebo <28 weeks gestation to 18 months post-birth	∅ 1 + 2 (MVMs only)	13/21

Parsons et al. [21]	264 children (Australia)	3 (At 7.5 yrs)	Strengths and Difficulties Questionnaire	Iron vs. placebo 20 weeks gestation to birth	∅ 3	17/21
						
			Short Temperament Scale for Children			

Schmidt et al. [28]	276 infants (rural Indonesia)	1,2 (At 6 and 12 mo)	Bayley Scales of Infant Development	Iron + folic acid + vitamin A vs. iron + folic acid vs. LD iron + LD folic acid 20 weeks gestation to birth	∅ 1, 2	14/21

Tamura et al. [29]	355 children (Alabama)	1,2,4,5 (At 5.3 yrs)	Differential Ability Scales	Zinc vs. placebo 20 weeks gestation to birth	∅ 1,2,4,5	15/21
						
			Wide Range Achievement Test			
						
			Knox Cube test			
						
			Gross Motor Scale			
						
			Grooved Pegboard test			
						
			Peabody Picture Vocabulary Test			
						
			Visual Sequential Memory			
						
			Auditory Sequential Memory			

Tofail et al. [23]	249 infants (Bangladesh)	1,2,3 (At 10 mo)	Bayley Scales of Infant Development	Fish oil vs. soy oil 25 weeks gestation to birth	∅ 1,2,3	15/21
						
			Wolke's Behaviour Rating Scale			

Tofail et al. [24]	2116 infants (Bangladesh)	1,2 (At 7 mo)	Willatts' Infant Planning Test	Food packets + MVM vs. food packets + iron + folic acid (Fe60) vs. food packets + LD iron + folic acid (Fe30) <30 weeks gestation to birth	∅ 1,2	17/21
						
			Bayley Scales of Infant Development - Psychomotor Developmental Index			

Zhou et al. [30]	302 children (Australia)	1,3 (At 4 yrs)	Stanford-Binet Intelligence Scale Strengths and Difficulties Questionnaire	Iron vs. placebo 20 weeks gestation to birth	∅ 1,3	17/21

1 = cognitive development

2 = motor development

3 = behavioral development

4 = visual development

5 = auditory development

∅ = no effect of nutrient(s) on development

+ = positive effect of nutrient(s) on development

- = negative effect of nutrient(s) on development

MVM = multivitamin-mineral

HCHP = high calorie, high protein

LCLP = low calorie, low protein

MVM-A = multivitamin-mineral without vitamin A

MVM+A = multivitamin-mineral with vitamin A

LD = lower dosage

Given the heterogeneity of methods and outcome measures in the studies reviewed, it was not feasible to compare effect sizes or to perform a meta-analysis.

### Study Characteristics

In general, the variability in this small set of papers was unexpectedly high in virtually every dimension evaluated. The 18 studies reviewed were published from 1983 to 2010, and included pregnant women from 14 different countries (ranging from Australia to Tanzania), varying across rural and urban settings in developing and industrialized nations (Table 1). Sample size varied from 29 to over 2000 participants. The offspring were assessed from two months to nine years age.

The interventions (Table 2) ranged from a single micronutrient (four studies) [21,22,29,30] to multi-micronutrients of various combinations (six studies) [13,24-28], as well as n-3 fatty acids in the forms of cod liver oil (two studies) [15,16], blended fish oil (three studies) [20,23,31], docosahexaenoic acid (DHA) in capsules (one study) [19], and DHA-containing cereal bars (two studies) [17,18]. Of the six multi-micronutrient formulas, no two were alike.

**Table 2:** Composition of (daily) micronutrient, macronutrient and fatty acid interventions in studies included in the systematic review

	Vit. A (mcg RAE)	B_1 _(mg)	B_2_(mg)	B_3 _(mg)	B_6 _(mg)	B_12 _(mcg)	Folic acid (mg)	Vit. C (mg)	Vit. D (IU)	Vit. E (mg)	Iron (mg)	Zinc (mg)	Cu (mg)	I (mcg)	Se (mcg)	DHA (g)	EPA (g)
Caulfield et al. [13] "zinc + iron + folic acid"	-	-	-	-	-	-	250	-	-	-	60	25	-	-	-	-	-

Caulfield et al. [13] "iron + folic acid"	-	-	-	-	-	-	250	-	-	-	60	-	-	-	-	-	-

Dunstan et al. [31]	-	-	-	-	-	-	-	-	-	12	-	-	-	-	-	2.2	1.1

Hamadani et al. [22]	-	-	-	-	-	-	-	-	-	-	-	30	-	-	-	-	-

Helland et al. [15,16]	1170	-	-	-	-	-	-	-	400	14	-	-	-	-	-	1.2	0.8

Innis & Friesen [19]	-	-	-	-	-	-	-	-	-	-	-	-	-	-	-	0.4	-

**^a,b^**Joos et al. [25] "HCHP drink"	1500	1.6	1.8	20.0	1.6	2.0	-	75	400	6.7	12	-	1.0	-	-	-	-

**^b^**Joos et al. [25] "LCLP drink"	*	*	*	*	*	*	*	*	*	*	*	*	*	*	*	-	-

**^c^**Judge et al. [17,18]	-	-	-	-	-	-	-	-	-	-	-	-	-	-	-	0.2	-

Li et al. [26] "MVM"	800	1.4	1.4	18	1.9	2.6	0.4	70	200	10	30	15	2.0	150	65	-	-

Li et al. [26] "iron + folic acid"	-	-	-	-	-	-	0.4	-	-	-	60	-	-	-	-	-	-

Li et al. [26] "folic acid"	-	-	-	-	-	-	0.4	-	-	-	-	-	-	-	-	-	-

Malcolm et al. [20]	-	-	-	-	-	-	-	-	-	-	-	-	-	-	-	0.2	-

**^d^**McGrath et al. [27] "vitamin A"	6000	-	-	-	-	-	-	-	-	-	-	-	-	-	-	-	-

McGrath et al. [27] "MVM-A"	-	20	20	100	25	50	0.8	500	-	30	-	-	-	-	-	-	-

**^d^**McGrath et al. [27] "MVM+A"	6000	20	20	100	25	50	0.8	500	-	30	-	-	-	-	-	-	-

Parsons et al. [21]	-	-	-	-	-	-	-	-	-	-	20	-	-	-	-	-	-

Schmidt et al. [28] "iron + folic acid + vitamin A"	4800	-	-	-	-	-	0.5	-	-	-	120	-	-	-	-	-	-

Schmidt et al. [28] "iron + folic acid"	-	-	-	-	-	-	0.5	-	-	-	120	-	-	-	-	-	-

Schmidt et al. [28] "LD iron + LD folic acid"	-	-	-	-	-	-	0.25	-	-	-	90	-	-	-	-	-	-

Tamura et al. [29]	-	-	-	-	-	-	-	-	-	-	-	25	-	-	-	-	-

Tofail et al. [23]	-	-	-	-	-	-	-	-	-	-	-	-	-	-	-	1.2	1.8

**^e^**Tofail et al. [24] "food + MVM"	800	1.4	1.4	18	1.9	2.6	0.4	70	200	10	30	15	2	150	65	-	-

**^e^**Tofail et al. [24] "food + Fe60"	-	-	-	-	-	-	0.4	-	-	-	60	-	-	-	-	-	-

**^e^**Tofail et al. [24] "food + Fe30"	-	-	-	-	-	-	0.4	-	-	-	30	-	-	-	-	-	-

Zhou et al. [30]	-	-	-	-	-	-	-	-	-	-	20	-	-	-	-	-	-

*Nutrient content not quantified in study protocol

Additional compositions:

^a^Joos et al. [25] - calcium 1000 mg, phosphorus 800 mg, sodium 400 mg, potassium 1.8 g, manganese 2 mg, fibre 1.1 g, 800 kcal, protein 40 g

^b^Joos et al. [25] - additional vitamins and minerals provided by multivitamin-mineral tablet were unspecified

^c^Judge et al. [17,18] - 70 kcal, protein 1 g, carbohydrate 15 g

^d^McGrath et al. [27] - vitamin A dose provided as 30 mg beta-carotene (4500 mcg RAE) and 5000 IU (1500 mcg RAE) preformed vitamin A

^e^Tofail et al. [24] - quantity of daily protein provided by food packets was unspecified

Abbreviation definitions:

Vit. = vitamin

B1 - Thiamine

B2 - Riboflavin

B3 - Niacin

B6 - Pyridoxine

B12 - Cobalamine

Cu - Copper

I - Iodine

Se - Selenium

Exposure dose and time period also varied considerably, although there was greater agreement about treatment onset: most began prior to 26 weeks gestation. Several studies failed to specify the rationale used for the selection of the ingredients of their formula or the dose [22].

Follow up period also varied greatly among studies, from 60 days to 66 weeks for visual acuity tests, and 3 months to 7.5 years for developmental tests. A number of studies had repeated follow up time points where assessment was conducted. Some studies showed a positive outcome at one assessment time, and null outcome at another time [18,26,27]. Outcome did not appear to be associated with length of follow up period.

### Quality of Reporting

The total scores on the CONSORT checklist ranged from 12 [16,17] to 19 [26], with a mean score of 14.95 ± 1.80 (the maximum possible score was 21). The median score was 15. Four studies had quality scores rated "good", 12 were rated "average", and two were "poor". The agreement between the pair of reviewers who independently assessed the RCTs using the CONSORT checklist was excellent [32] (ICC = 0.775; 95% CI = 0.491-0.910; P < 0.001).

The majority of studies did not meet the CONSORT statement standards [14], and none of the studies reported all 21 items assessed. For example, only seven studies (38.9%) reported how sample size was determined, one of which determined sample size post hoc. While all of the studies reviewed were reported as RCTs, only 33% (n = 6) provided information on generation of the random allocation sequence, 22% (n = 4) referred to concealment of the allocation sequence, and 11% (n = 2) described the implementation of the randomization. Furthermore, only Judge et al. [17] and Innis and Friesen [19] reported on all three items relating to randomization, whereas nearly 70% (n = 12) of the RCTs reviewed did not report any information regarding randomization. Information on blinding after intervention assignment was somewhat better reported, yet 38.9% (n = 7) of the studies still failed to mention who was blinded to group assignment.

Of the four studies that rated "good" for quality, one study on multivitamin-mineral supplementation reported a positive outcome [26], and the other three reported no effect [21,24,30]. Two studies on fish oil/DHA that rated "poor" on quality [16,17] did report positive outcomes. Thus, there was no consistency between study quality and outcome.

Seven studies with a positive outcome had quality scores between 12 to 19 [16-18,25-27,31]. Four of these studies had fish oil/DHA as the intervention, and three were multivitamin-minerals. Eleven studies had null or negative outcomes, with quality scores ranging from 14 to 17. In these 11 studies, six used a single micronutrient, four used fish oil/DHA, and one used multivitamin-mineral food packets. Furthermore, 10 studies were conducted in Western "developed" countries, of which four had positive outcomes and six had null/negative outcomes; eight studies were from "developing" countries in Asia, Eastern Europe, South America, and Africa, of which four had positive outcomes and four had null/negative outcomes. Quality scores for studies in "developed" countries ranged from 12 to 17, while for "developing" countries, the range was 13 to 19. Outcome did not appear to be associated with location of the study.

### Mental Development Outcomes

Three trials reported improvements in cognitive development, each with different single and multi-nutrient supplements [16,17,26], yet the improvements were transient with respect to age groups tested in two of the studies. There was no consistency among these reports regarding the specific aspect of cognitive development that improved, whether processing, problem solving or overall cognitive development.

Psychomotor outcomes were improved in three studies: two involving multivitamin-mineral supplements [25,27], and one with fish oil capsules [31]. Both the multivitamin-mineral supplement studies found improved psychomotor scores using the Bayley Scales of Infant Development, whereas the study on fish oil [31] found improvements in hand-eye coordination.

There were no trials reporting significant differences between treatment and placebo groups among those assessing behavior or temperament. Visual development was improved in one study using DHA supplementation [18]; however, improvement in visual acuity was seen at four but not at six months of age. All other studies investigating visual development had null results.

Negative effects on developmental outcomes were reported in a small number of studies. Hamadani et al. [22] found that children whose mothers were supplemented with zinc prenatally scored lower on cognitive and psychomotor indices. Similarly, two studies which examined the effects of iron on neurodevelopment [21,30] found higher, but non-significant, rates of abnormal behavior and peer problems in iron-supplemented groups compared with placebo, though this was only in a small subsample from each study.

## Discussion

### Principal Findings

This systematic review revealed no conclusive evidence that an enhanced intrauterine environment through nutrient supplementation was associated with better mental development in the child. While cognitive, psychomotor and visual function showed improved outcomes with supplementation in several studies, the findings were often transient (not detectable when children were tested later in life) with poor corroboration among studies.

Though not conclusive, there was some evidence to support supplementation with n-3 fatty acids or multiple (but not single) micronutrients having some positive effect on mental development. Among the n-3 fatty acid intervention studies, four reported higher scores in one or more outcome measure in the intervention group compared to the placebo group [16-18,31], while four studies found no difference between the groups [15,19,20,23]. There were significant methodological limitations in some of the studies with no results, which may be responsible for the null findings. For instance, there was poor compliance with the n-3 fatty acid capsules in the study by Tofail et al. [23], and there was significant contamination in the 'placebo' group used by Helland et al. [15,16], in that approximately 50% of children, regardless of group assignment, consumed cod liver oil regularly during the preschool years. Also, the dose and formulation employed by two of the studies [19,20] was relatively low at 200 mg of only DHA.

There is epidemiologic evidence that maternal dietary n-3 fatty acid intake influences mental development. For example, Gale et al. [33] used the Strength and Difficulties Questionnaire and Wechsler Abbreviated Scale of Intelligence to examine the association between fish intake in pregnant women and their children's IQ at age 9. Compared to mothers who did not eat oily fish during gestation, there was a reduced risk of hyperactivity in children whose mothers had eaten oily fish in early pregnancy and higher verbal IQs in those whose mothers ate fish in late pregnancy

Among the vitamin and mineral studies reviewed here, better cognitive and/or psychomotor outcomes were reported for supplementation when the intervention involved multiple micronutrients [25-27]. In contrast, the results for single micronutrient supplementation of pregnant women on child neurodevelopment were null for iron [21,30], folic acid [28], vitamin A [28], and zinc [13,29]. The positive findings with respect to multi-nutrient supplementation are supported in the literature. Wehby and colleagues [34], using survey data, found that prenatal multivitamin-mineral use was associated with reduced risk of language and social development delays during childhood, whereas single micronutrients showed variable effects, several of which were negatively associated with developmental outcomes.

Other correlational and epidemiologic studies of maternal nutrient status and child development have reported mixed and inconsistent results. Bhate et al. [35] did a follow-up comparison of cognitive function in 9-year-old children, and found that children of women with high plasma B_12 _during pregnancy performed significantly better on the Color Trail Test (subtest A) and Digit Span Test (backward), but not on the Raven's Colored Progressive Matrices and Visual Recognition tests. However, another follow-up study compared cognitive development in 5-year-old children as a function of maternal folate status during pregnancy, finding no differences in the test scores of neurodevelopment between the low folate and normal folate groups [36].

Given that maternal nutrient status and intake appear to be associated with infant outcomes in terms of physical health, and perhaps some indicators of mental function, should broader supplementation guidelines be considered? A number of studies have found nutrient inadequacies in pregnant women living in western countries consuming a typical western diet [37]. A study by Ray et al. [38] reported that after a decade of folic acid fortification, other B-vitamin deficiencies, such as B_12_, continue to occur in up to five percent of pregnant women. These authors concluded that B_12 _deficiency may be an independent risk factor for neural tube defect. Thus, even in developed, nutritionally-abundant countries, nutrient inadequacy or deficiency may be more common than realized.

Another aspect of nutrient requirement not considered is genetic variance. A report by Cavalli et al. [39] discussed the "folic resistance" hypothesis among some women. That is, while prenatal folic acid supplementation prevented about 70% of neural tube defects in one dataset [40], there are cases of folate-resistant and folate-sensitive NTD subtypes, which are supported by animal models [39]. In a case series, Cavalli and colleagues [39] supplemented women with high NTD recurrence risk with periconceptional inositol and folic acid to prevent reputed folate-resistant fetal NTDs. The addition of inositol to folic acid appeared to prevent the recurrence of NTDs in subsequent pregnancies and deliveries. Thus, this is further evidence that multi-micronutrient supplementation during pregnancy may confer greater benefit than single nutrient supplementation for infant outcomes.

### Limitations of Current Evidence

The primary limitations were related to the methodologies and reporting of the 18 studies included in this review: unclear recruitment, randomization, blinding, follow up, unclear or unspecified supplement dosage and reason for dosage setting, and lack of monitoring or reporting of compliance. Other limitations we noted were small sample size, variability of follow-up periods, and inadequate information regarding factors such as home environment, education, and level of stimulation. These social and educational variables are important in that the children were exposed to them between birth and the time of final assessment, hence influencing outcomes. Another design deficiency across the studies is the lack of measurement for dietary intake and/or nutrient status of the women prior to supplementation. This information would be a valuable baseline for assessing whether the adequacy of women's intake or status was relevant to supplementation outcomes.

There is also some question regarding the selection of placebos used in the n-3 fatty acid intervention studies. While a true placebo is biologically inert, n-3 fatty acid intervention studies provide the control groups with metabolically-active compounds in order to maintain visual appearance, namely oils with varying concentrations of fatty acids. One could argue that these oils cannot serve as a control supplement given their distinct effect on maternal lipid and fatty acid profiles [41]. This use of metabolically active 'placebos' leads to uncertainty as to whether differences reported between n-3 fatty acids and 'placebo' groups can be attributed solely to increased n-3 fatty acid intake.

Few studies provided information pertaining to diet quality, whether by assessing nutrient status with blood samples or dietary intake through recalls or questionnaires. Thus, the adequacy of prenatal nutrient status was not known, limiting conclusions about how baseline dietary intake and status may affect infant mental development in supplementation trials. It is possible that if a woman has widespread nutrient deficiencies, supplementing with a single nutrient would not have any noticeable impact on the offspring's mental development, perhaps accounting for why multi-micronutrient supplements appeared to be associated with better outcomes.

Given the variability in study populations, interventions used, outcomes measured, and the overall low reporting quality of the studies, our findings cannot answer our a priori question nor be generalized to a broader population. This inconsistency among the studies is reflected in the low CONSORT scores. The use of the CONSORT checklist to evaluate studies may not always provide an adequate appreciation of rigor, because low scores may be at least partly explained by the historical focus of CONSORT - pharmaceutical and treatment-based RCTs; thus, the applicability of CONSORT to RCTs of a non-pharmaceutical nature is unclear. However, the use of a checklist like the CONSORT provided a means to assess consistency of the studies in this systematic review, and highlighted a number of limitations that made interpretation problematic.

### Strengths and Weaknesses of the Systematic Review

The initial search criteria employed here included only English language articles, and RCTs, pseudo-experimental and cohort studies, which may have resulted in missing some pertinent studies. Since much of the literature on the topic of gestational nutrition emerges from the developing world, the loss of some of the non-English literature may limit the generalizability of the conclusions that were drawn. Another limitation is publication bias: studies with null result may be less likely to be published, and would not be included in this review. The fact that a meta-analysis was not feasible is also a weakness that could not be overcome given the present status of the published research on this topic. On the other hand, this systematic review appears to be the first attempt to evaluate objectively the literature that is beginning to develop on the topic of prenatal supplementation and children's mental outcomes. Although many of the studies were published prior to the development of current methodologic standards (e.g., the CONSORT guidelines) and hence cannot be faulted for the weaknesses they manifest, the tabular presentation of those weaknesses at this point in time may be useful for guiding future studies in this area.

## Conclusions

This review attempted to assess the state of evidence for the relationship between prenatal supplementation and infant mental development. We recognize that infant mental development is the result of complex multifactorial processes. Nutrients form the bases for proper neural development and could have long-lasting impact on mental development later in life. Given that pregnant women are often told by primary clinicians to incorporate folate (with or without B_12_) and/or iron into their diet, it is important to know that the research seems to indicate that single nutrient supplementation is less adequate than supplementation with more complex formulas. This finding was derived from studies from both developed and developing countries. Although not conclusive at this stage, the evidence suggests there is value in further research examining the potential benefit of prenatal multi-micronutrient and n-3 fatty acid supplementation for child mental development. Future studies should consider the timing, duration, and required dosage of supplementation that meets the needs of the developing foetus to fully examine the impact of multi-micronutrients (including n-3 fatty acid) on child mental development.

## Competing interests

No potential conflict of interest was reported by the authors.

## Authors' contributions

All authors have made significant contributions to this study and meet criteria for authorship. All authors have read and approved the final manuscript.

## Pre-publication history

The pre-publication history for this paper can be accessed here:

http://www.biomedcentral.com/1471-2393/11/12/prepub
